# Clinical Evaluation of the Retention of Different Pit and Fissure Sealants: A 1-Year Study

**DOI:** 10.5005/jp-journals-10005-1215

**Published:** 2013-10-14

**Authors:** Parvathy Kumaran

**Affiliations:** Reader, Department of Pedodontics and Preventive Dentistry Amrita School of Dentistry, Kochi, Kerala, India, e-mail: parvathyravi@yahoo.co.in

**Keywords:** Retention, Pit and fissure sealant, Glass ionomer

## Abstract

**Objective:** The aim of this study was to evaluate the retention of different pit and fissure sealants on the first permanent molars over a period of one year***.***

**Materials and methods:** In this study, a total of 40 children with all first permanent molars erupted received four different pit and fissure sealants. The children were evaluated at 6 and 12 months.

**Results:** The data was subjected to Chi-square test and Kaplan Meier survival analysis. The p-value was calculated using Wilcoxon matched-pairs signed-rank test.

**Conclusion:** The retention rates of resin-based sealants were superior to that of glass ionomer sealant.

**How to cite this article:** Kumaran P. Clinical Evaluation of the Retention of Different Pit and Fissure Sealants: A 1-Year Study. Int J Clin Pediatr Dent 2013;6(3):183-187.

## INTRODUCTION

In modern dentistry, the importance of prevention is ever increasing. Preventive and therapeutic treatment based on the philosophy of health promotion in dentistry can interfere with the demineralization of dental tissue by arresting, balancing or decreasing the progression rate of carious lesions.^1^

The special topography of fissures makes the mechanical removal of plaque bacteria difficult thus accounting for the reduced efficacy of these preventive measures.^2^ Pits and fissures are eight times as vulnerable as smooth surface for dental caries. Consequently, the utilization of an occlusal barrier which isolates the occlusal surface from the surrounding environment in order to impede the onset of caries resulted in the emergence of the sealant systems.^3,4^

However, in spite of the proven efficacy and relative ease of application of sealant materials, retention is the main determinant in maintaining a sealant's caries-preventive effect.^5,6^ The retention of sealants relies upon the ability of the resin sealant to thoroughly fill pits and fissures and/ or morphological defects and remain completely intact and bonded to enamel surface for a life time.^3^

Thus, a clinical trial was undertaken to evaluate the retention rates of four different pit and fissure sealants on the first permanent molars over a period of 12 months.

## MATERIALS AND METHODS

Forty children, who reported to the Department of Pedodontics and Preventive Dentistry, College of Dental Surgery, Saveetha University, in the age group 7 to 10 years were selected based on a double-blinded analysis for fully erupted first permanent molars with intact deep and retentive fissures. Physically/mentally challenged children, those with poor systemic health under medication and children with deleterious oral habits affecting occlusion were excluded from the study.

The four sealants that were studied included (a) Clinpro^®^ (3M, St Paul, MN)*,* (b) Delton^®^ FS+ (Dentsply International, York, PA)*,* (c) Helioseal F ^®^ (Ivoclar Vivadent, NY) and (d) Fuji VII GIC^®^ (GC Corporation, Tokyo, Japan).

A thorough dental history was taken prior to our examination and the teeth were checked for caries or gingival disease with a no. 4 plain mirror and a no. 6 right-angled probe. The nature and objectives of the trial as well as the possible discomforts and benefits were explained, and an informed consent was obtained from the guardian.

The four permanent first molars of each child were randomly assigned for placement of each of the four materials after prophylaxis and polishing using a polishing brush/cone under running water in a slow speed hand piece to remove salivary pellicle and the remaining dental bio-film. The teeth planned for placement of the sealant were finally isolated using rubber dam and low volume suction throughout the procedure.

All the four sealants were applied based on manufacturers' instructions. The light curing was done using Q-LUX curing lamp (12V-75W, Rolence Enterprise Inc, Taiwan) initially for 20 seconds and extended by an additional time of 10 seconds to ensure complete polymerization of the sealant. A probe was run over the sealed surface to ensure the marginal seal between the sealant and the tooth surface.

After curing, the rubber dam was removed. Then the occlusion was checked with a carbon marker and premature contacts were relieved to ensure that the sealants do not produce any occlusal interference. Blinding was not possible as Fuji VII was visually distinct from the other sealant materials used.

All the cases were clinically evaluated after 6 and 12 months of application. The retention rate was assessed based on the criteria proposed by Tonn and Ryge (1982).

The examination results were categorized into three groups as follows:

- TR: Total retention–total retention of the sealant on the occlusal surface (score 0).
- PL: Partial loss–presence of sealant with fractures and loss of material (score 1).
- TL: Total loss–absence of the sealant on the occlusal surface (score 2).

The values obtained were then subjected to statistical analysis using SPSS software version no.15.

## RESULTS

Analysis of data was carried out using the chi-square test which was used to assess quantitatively whether or not the observed frequencies differ significantly from those expected on the basis of a null hypothesis.

Of the 40 children provided with sealants at baseline, all the 40 returned at 6-month recall appointments and only 38 reported at the 12-month recall.

**Graph 1:**
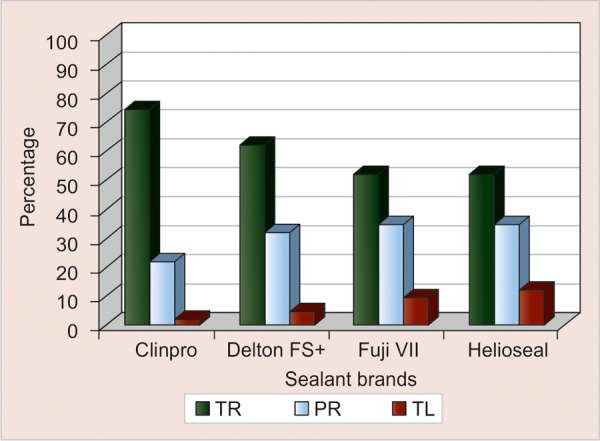
Cross tabulation of 6 months outcome by material

**Graph 2:**
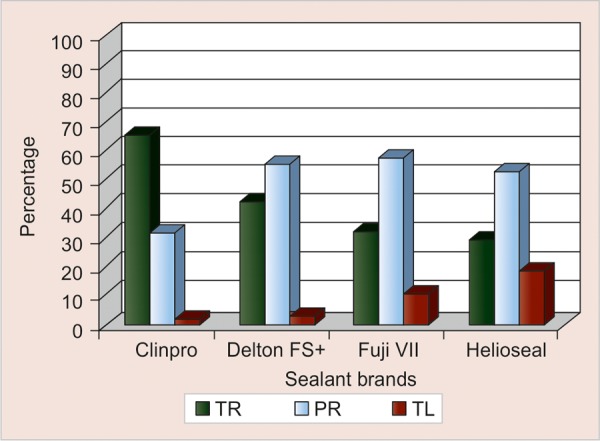
Cross tabulation of 12 months outcome by material

Table 1, Graphs 1 and 2 show the cross tabulation of the rate of retention of the four sealants at 6 and 12 months.

At 6 months, Clinpro showed 75% total retention, 22.5% partial retention and 2.5% total loss. Around 62.5% was totally retained for Delton FS+, 32.5% partially retained and 5% totally lost. Similar retention rates noticed for Helioseal F and Fuji VII were 52.5, 35, 12.5 and 52.5, 37.5 and 10% respectively.

Chi-square test was used to determine the outcome of the four sealants at 6 months. A p-value of 0.30 indicated that the difference in the retention rates of the different sealants was insignificant.

**Graph 3:**
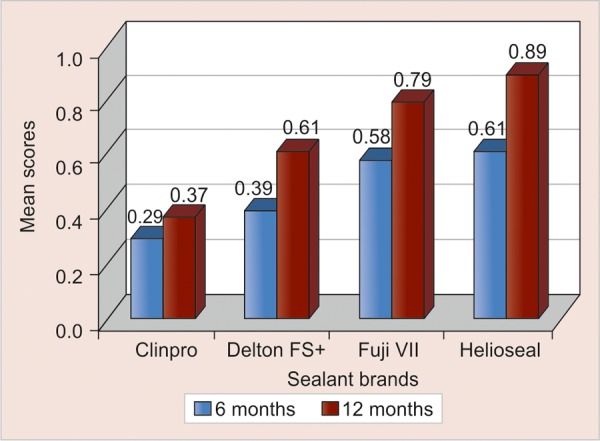
Comparison of the mean scores of the different sealants at 6 and 12 months

The 12-month results for Clinpro were 65.8% total retention, 31.6% partial retention and 2.6% total loss. In comparison, Delton FS+ was 42.1% totally retained, 55.3% partially retained and 2.6% totally lost. Helioseal F showed the highest rates of total loss of 18.4% followed by Fuji VII with 10.5%. The differences in the retention rates were significant at the 12-month recall with a p-value of 0.005. Table 2 and Graph 3 show the comparison of the mean scores between 6 and 12-month for each of the four sealants.

**Table 1:** **Table 1:** Cross tabulation of 6 and 12 months outcome by material

*Sealants*		*TR*				*PR*				*TL*		
		*6 months*		*12 months*		*6 months*		*12 months*		*6 months*		*12 months*
Clinpro		75%		65.8%		22.5%		31.6%		2.5%		2.6%
Delton FS+		62.5%		42.1%		32.5%		55.3%		5%		2.6%
Fuji VII		52.5%		31.6%		37.5%		57.9%		10%		10.5%
Helioseal F		52.5%		28.9%		35%		52.6%		5%		18%

**Table 2:** **Table 2:** Comparison of mean scores between 6 and 12 months for each material

*Sealants*		*6 months Mean ± SD*		*12 months Mean ± SD*		*Change Mean ± SD*		*p-value*
Clinpro		0.29 ± 0.52		0.37 ± 0.54		0.08 ± 0.27		0.11 NS
Delton FS+		0.39 ± 0.55		0.61 ± 0.55		0.21 ± 0.41		0.01 S
Fuji VII		0.58 ± 0.68		0.79 ± 0.62		0.21 ± 0.41		0.01 S
Helioseal F		0.61 ± 0.72		0.89 ± 0.69		0.29 ± 0.46		0.003 S

NS: Nonsignificant; S: Significant

For Clinpro, the mean score at 6 months was 0.29 ± 0.52 which was increased to a mean score of 0.37 ± 0.54 at 12 months. Thus, there was a mean increase of 0.08 ± 0.27 which was not statistically significant (p - 0.11).

However for Delton FS+, Fuji VII and Helioseal F the increase in the mean scores were 0.21 ± 0.41, 0.21 ± 0.41 and 0.29 ± 0.46 respectively which were statistically significant (p < 0.01).

Wilcoxon matched-pairs signed-rank test was used to calculate the p-value. Clinpro did not show a significant difference in the mean scores at the two time periods (p < 0.11) indicating that its performance was more or less similar at 6 and 12-month.

Table 3 shows the comparative survival pattern of the four sealants. Kaplan-Meier survival analysis was done to compare the survival pattern of four different sealant brands. Log-rank test was used to test the significance of survival curves among the materials.

Mean survival time for Clinpro was 10-month with a 95% confidence interval of 10 to 12 months; whereas for Delton FS+, Fuji VII and Helioseal F, mean survival was 10-month with a 95% confidence interval of 9 to 10 months.

Significance test by long-rank test showed that there is a significant difference between Clinpro and Fuji VII (p < 0.003) and Clinpro and Helioseal F (p < 0.005) with regard to their survival pattern.

**Table 3:** **Table 3:** Kaplan-Meier survival analysis

Sealants		Mean ± SE (95% CI)		Overall p-value
Clinpro		10 ± 0 (10-12)		
Delton FS+		10 ± 0 (9-10)		
Fuji VII		10 ± 0 (9-10)		0.01 S
Helioseal F		10 ± 0 (9-10)		

S: Significant

## DISCUSSION

In this era of preventive dentistry, arrays of dental materials are available oriented toward primary prevention of dental diseases but the complex morphology of the occlusal surface in the form of pit and fissure jeopardizes mechanical plaque removal and proven preventive measures thus asking for specific prevention of occlusal caries.^6^ Considering the fact that occlusal surfaces constitute only 12% of the tooth surface, they are eight times as vulnerable as smooth surfaces to caries.^3^ So, prevention of occlusal caries assumes paramount importance in the preservation of tooth structure.

So, maintenance of oral hygiene in conjunction with fluoride therapy and prudent use of pit and fissure sealant seems to be the best strategy in preserving the integrity of the tooth structure.^7^ Muller-Bolla et al in a systematic review stated that the notion of retention is capital because the main function of sealants is to change pit and fissure morphology to form an efficient physical barrier between the enamel surface and oral environment for as long as possible. Thus, the complete retention of the sealant associated with duration is the principal clinical evaluation criteria now used as a surrogate measure of effectiveness in preventing decay.^8^

Previous studies have proved that the most critical period for sealant failure is at baseline and during the 6 months following application. Thus the study was undertaken for 12 months. However, a longer follow-up period would definitely give more confidence about the results.^9^

The most appropriate period for the placement of occlusal sealants is soon after eruption of the permanent molars, because recently erupted teeth are less mineralized than those exposed to oral environment for several years. Such teeth have also not undergone the benefits of post eruptive maturation of the enamel and may be thus more prone to acid attack. In such conditions, early placement of sealants may prevent the development of carious lesions on occlusal pits and fissures.^10^ This explains that the age group of 7 to 10 years selected in this study was appropriate for sealing the first permanent molar.

The isolation of the tooth from contamination by saliva is one of the most important aspects of sealant placement because the total clinical procedure corresponds to a technique which is sensitive in that saliva contamination after the acid etching stage prevents the formation of tags and thereby the mechanical retention of the resin.^11^

Several methods of cleaning the fissures have been advocated over the years, but it appears that all are relatively equal in the results they obtain.^12^ Keeping this in view in the present study, the occlusal surface of each tooth was cleaned with a blunt probe, washed and dried with a three-in-one syringe.

One of the reasons for the better performance of Clinpro may be due to the fact that it is an unfilled sealant. This is supported by Rock et al who using a split mouth design concluded that an unfilled light-cured resin-based sealant was significantly better retained than a filled one. An unfilled resin would penetrate deeper into the fissure system because of its lower viscosity and therefore would, perhaps, be better retained.^13^ This fact was also ascertained by Percinoto et al who reported that the fillers increase the viscosity and that the viscosity of the sealant materials effected the penetration into the microporosities created by acid etching which is a prerequisite for sealant placement.^14^

Another reason may be that Clinpro presents with a unique property of color change–from the original color of pink to white on being light cured. The pink color allows for the better visualization of the sealant in the fissures which would ensure that the pits and fissures are completely covered with the sealant.^3^

The results for Delton FS+ were quite encouraging. There was no significant difference between the mean scores of Clinpro and Delton FS+. The probable reason could be that Clinpro was unfilled and Delton FS+ is a semifilled sealant with a low viscosity.

Delton FS+ is a sealant in which sodium fluoride is added to the unpolymerized resin in the form of a soluble fluoride salt. According to the National Institute of Dental Research, the fluoride release takes place by the dissolution of the soluble salt which might in turn weaken the sealant in situ, thus reducing its usefulness as a preventive agent.^15^

A significant difference was found between the mean scores of Fuji VII and Clinpro after 12-month (p < 0.003). It was seen from the performance of Fuji VII that there is a gradual loss in the retention of the material during the follow-up periods in contrast to that shown by Clinpro. Clinpro showed an initial loss of retention during the 6-month evaluation, but later on got stabilized without much appreciable change in the retention of the material.

According to few investigators, a relevant factor that should be considered when glass ionomer material is being used as a sealant material is that even after it has been clinically lost, small amount of sealants will be left at the bottom of the fissures and continue to release fluoride. So, in spite of its partial loss, protection is still afforded to the tooth structure, and fluoride plays a pivotal role in enamel remineralization, thus reducing the susceptibility to future decay in contrast to resin-based sealant which makes the tooth more susceptible to caries after sealant loss.^16^

Another important aspect to be noted was that the resin sealant appeared to be lost in bulk, leaving behind uneven margins. In contrast, the glass ionomer sealant usually did not develop marginal discrepancies but did appear to wear excessively. The lower retention rate for the glass ionomer may be attributed to the lower compressive and tensile strengths of the glass ionomer sealant and not a lower abrasion resistance.^17,18^

Helioseal F applied in this study contained fluorosilicate glass fillers. The low retention rate of Helioseal F is based on the rapid formation of calcium fluoride in very small particles form that inhibits Helioseal F's sealing capability onto the enamel surface.^14^ The very presence of fillers also accounts for a higher viscosity thereby decreasing the penetrability of Helioseal F.

## CONCLUSION

The retention of the resin-based sealants were superior to that glass ionomer cements. But, the real efficacy of glass ionomer sealants still remains open, warranting the need for further clinical trials. So, through judicious use of dental sealants, we can effectively alter the morphology of the tooth and remove one of the proven ecological niches of *Streptococcus mutans*, thus achieving our goal of primary prevention and preserving the integrity of the dentition.
